# Hypospadias: A Comprehensive Review Including Its Embryology, Etiology and Surgical Techniques

**DOI:** 10.7759/cureus.27544

**Published:** 2022-07-31

**Authors:** Sattam A Halaseh, Shahed Halaseh, Mohannad Ashour

**Affiliations:** 1 General and Colorectal Surgery, Torbay Hospital, Torbay and South Devon NHS Foundation Trust, Torquay, GBR; 2 Surgery, Jordan University Hospital, Amman, JOR; 3 Urology, The Specialty Hospital, Amman, JOR

**Keywords:** pediatric suregery, outcome, external genital development, penis, hypospadias, hypospadias repair

## Abstract

Hypospadias is among the most prevalent urogenital malformations in male newborns. It is characterized by the displacement of the urethral meatus to the ventral side of the penis, an aberrant ventral curve of the penis referred to as "chordee," and an abnormally arranged foreskin with a "hood" found dorsally and lacking foreskin ventrally. Patients may have an extra genitourinary abnormality based on the area of the lesion. In around 70% of cases, the urethral meatus is positioned distally to the shaft, representing a milder form of the disease. The remaining 30% of cases are located proximally, are more complicated, and require further evaluation. Although the origin of hypospadias is mostly obscure, several suggestions exist about genetic susceptibility and hormonal factors. The objective of hypospadias restoration is to restore aesthetic and functional regularity, and surgery is currently advised at a young age, mostly between six and 18 months. At any age, hypospadias can be repaired with an equivalent risk of complications, functional outcomes, and aesthetic outcomes. However, the best age of treatment is still undetermined. Even though the long-term effects on appearance and sexual function are usually good, males may be less likely to make the first move after rectification. Also, people who have hypospadias treated are twice as likely to have problems with their lower urinary tract. These problems can last for years after the initial repair.

## Introduction and background

**Figure 1:**
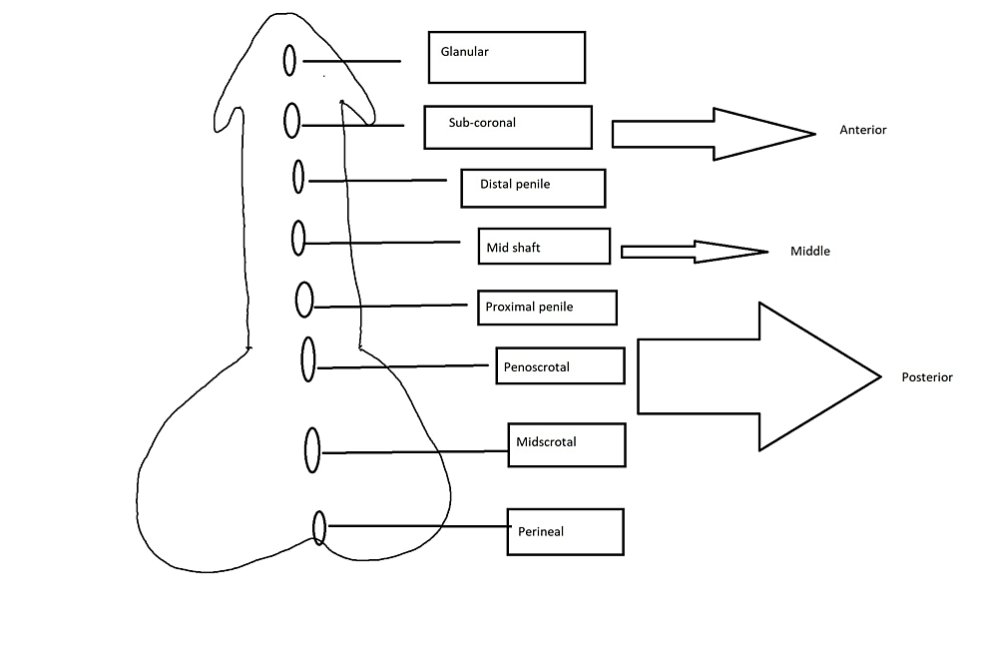
Duckett classification of hypospadias.

Hypospadias is a congenital deformity of the external genitalia in males. It is defined by the aberrant growth of the urethral fold and the ventral foreskin of the penis, which results in the incorrect location of the urethral opening [1]. In hypospadias, the external urethral meatus may be mispositioned to a different degree and may be accompanied by penile curving. Patients could have an extra genitourinary abnormality based on the location of the hypospadias [2,3]. It is considered among the most prevalent congenital abnormalities in males. Hypospadias occurs in one out of 150 to 300 live births [4,5]. After undescended testis, hypospadias is the second most common congenital abnormality [2]. Hypospadias is frequently characterized as posterior, penile, or anterior based on the preoperative location of the meatus. Nearly 70% of hypospadias are glandular or distally placed on the penis and are regarded as moderate variants, while the remaining are more severe and complicated. This classification was suggested by Duckett [6] (Figure 1).

The standards are used to define and evaluate hypospadias. Meatal position alone is widely regarded as a rudimentary method for classifying the severity of hypospadias since it does not consider the degree of tissue abnormality. In addition, the size of the penis, the size of the glans and urethral plate, the amount of separation of the corpus spongiosum, the existence of curvature, as well as abnormalities, and the location of the scrotum, have a substantial impact on the success of surgical correction. Consequently, a definitive classification can only be made following surgery [7].

This review article focuses on describing the embryological defects that cause hypospadias and the clinical characteristics of the condition. Outline the classification of hypospadias, its management options, the timing of surgery, and its results.

## Review

Embryology

When penile growth is halted, it produces the three-fold classic triad of hypospadias, which includes a hooded dorsal foreskin, an inverted penile curvature on the dorsal side of the foreskin, and a proximal urethral meatus.

In the earliest weeks of embryonic development, the abnormal or incomplete closure of the urethra is the primary pathophysiological event that leads to hypospadias. Development of the external genitalia happens in two stages, which for both sexes are identical. In the first phase, which occurs between the fifth and eighth weeks of pregnancy, the primordial genitalia is formed in the absence of a hormonal stimulus. During this phase, mesodermal cells oriented laterally to the cloacal membrane produce the cloacal folds. These folds combine anteriorly to create the genital tubercle (GT), then break posteriorly into the urogenital and anal folds that surround the urogenital sinus. The GT is composed of three cell layers: the lateral plate mesoderm, the surface face ectoderm, and the endodermal urethral epithelium. This is the primary signaling center for GT's development, differentiation, and outgrowth [8].

In men with chromosomes XY, the second phase, a hormone-dependent stage, begins with the development of gonads into testes. Two of the most significant actions of testicular testosterone are the elongation of the GT and the formation of the urethral depression. The urethral plate, the distal section of the urethral groove, is delineated laterally by the urethral pleats and extends into the glans penis. The urethra is generated when the urethral folds merge, and the coat of the penis is created from the outer surface of ectodermal cells, which merge with the ventral part of the phallus to form the median raphe.

Various malformations, including hypospadias, an abnormal curve of the penis, and improper penile foreskin generation, can be caused by genetic disruption or change of signaling pathways in male external genital and urethral development.

Epidemiology

Approximately 18.6 out of every 10,000 live births in Europe are affected by hypospadias. Registrations in 23 European registries between 2001 and 2010 demonstrated a steady number despite previously observed increases and decreases in temporal patterns [9]. North America has the highest prevalence, with 34.2 cases per 10,000 live births, whereas Asia has the lowest, at 0.6-69 cases per 10,000 live births. Even with more than 90 million screened newborns, the real global prevalence and trends are still difficult to quantify due to various methodological issues [5].

Given its frequency, hypospadias can place a significant strain on healthcare spending. A significant risk of complications may necessitate many procedures, particularly in the most severe instances. In addition, a substantial proportion of patients struggle with aesthetic or functional issues [2,10].

Etiology

Concerning the genesis of hypospadias, several explanations have been offered, including genetic susceptibility, insufficient prenatal hormone stimulation, maternal-placental variables, and environmental impacts. Thus, it is plausible that hypospadias has several causes [11]. Premature birth, small-for-gestational-age newborns who are less than the 10th percentile for weight, length, and/or head circumference, and intrauterine growth restriction are risk factors. All of these have been linked to an increased chance of having a baby with hypospadias [12,13] (Table 1). Hypospadias rates have been linked to both inadequate placentas and the use of assisted reproductive technologies [14,15].

**Table 1:** Risk factors for hypospadias.

Genetic abnormalities
Environmental exposure
Small for gestational age ( <10^th^ percentile for weight, length, and head circumference )
Intra-uterine growth restriction.

One in every seven occurrences of hypospadias is passed down through first, second, or third-degree family members. For anterior and middle forms, familial occurrence appears to be more prevalent than for posterior kinds. It is estimated that between 9 and 17% of the male siblings of a hypospadias-infected kid may get the condition [11]. One-third of hypospadias are directly linked to a genetic abnormality [16]. Nearly 200 disorders with recognized genetic etiology are connected with hypospadias. However, only a percentage of males with idiopathic variants have this condition [17]. The most common associations are WAGR syndrome, Denys-Drash syndrome, and Smith-Lemli-Opitz syndrome [2,18].

Another important factor in hypospadias is hormonal influence. Most hypospadias is solitary conditions, while uni-bilateral cryptorchidism and micropenis are related abnormalities [19]. These co-morbidities indicate a lack of hormonal effects during development. Androgens and estrogens both play a crucial role in genital development, and in the event of an imbalance, a range of congenital penile malformations, including hypospadias, micropenis, and ambiguous genitalia, can be observed [19]. A shortened anogenital distance in males with hypospadias as a consequence of a disturbance in embryonic androgen exposure [20] is a clinical observation that supports this notion. Other studies highlight the possible impact of so-called endocrine-disrupting environmental pollutants on the formation of hypospadias. Hypospadias was created in mouse models by the exposure of their mothers to synthetic estrogens. Due to the enormous variances across animals, it remains disputed whether someone has a significant effect on humans [21].

Evaluation

Hypospadias is among the most prevalent birth defects in males. A misplaced, ventrally-located urethral meatus; a ventral penile curvature; and an imperfect, dorsally-hooded foreskin are the physical exam criteria for diagnosing an ectopic urethral meatus. Hypospadias is a vast concept, however, and the degree of each symptom can vary significantly across boys. The second and third components are not usually present. Up to 5% of boys suffering from hypospadias have an undamaged prepuce, and the condition is not recognized till the foreskin becomes retractable or diminished during circumcision. Since an intact prepuce can conceal the existence of inadequate urethral growth in a newborn infant, it is essential to retract the foreskin before circumcision to prevent losing this oddity and presumably harming the imperfect urethra or expelling foreskin that could be incorporated into a subsequent urethral reconstruction [22].

Initial assessment of males with hypospadias must include a thorough medical history and physical examination. In conjunction with the trio of hypospadias, males may have related abnormalities such as penile torsion, penoscrotal webbing, and penoscrotal displacement, which must be taken into account while planning the surgery. On physical examination, boys with hypospadias may have dysplastic ventral tissue. On examination, a shortage of ventral axis skin may be instantly apparent.

The position of the urethral meatus has traditionally been used to determine the degree of hypospadias [7]. Using these criteria, almost 85% of males have a mild distal meatus variation [23]. Proximal hypospadias occurs in almost 15% of individuals and provides the surgeon with various distinct therapeutic issues [9].

A classification of hypospadias based only on the position of the urethral meatus is very simplistic and may even be deceptive. A classification system that incorporates the position of the urethral opening and the degree of penile curvature following degloving results in a more accurate and pertinent diagnosis.

The GMS score (glans meatus and penile shaft [curvature]) integrates physical exam outcomes in the operating room, evaluating the quality of the glans and urethral plate, the position of the urethral opening, and the degree of penile curvature, to objectively allocate scores for severity stratification (Table 2). The GMS score was designed for use in the operating room since office measures are less reliable in determining severity, namely the extent of ventral penile curvature [24,25].

**Table 2:** The glans meatus and penile shaft (GMS) score From Merriman et al., 2013 [25].

Glans score [G]
Good size : healthy urethral plate, deeply grooved Adequate size : adequate urethral plate, grooved Small in size: narrow urethral plate, fibrosis or flat Very small : indistinct urethral plate, very narrow or flat
Meatus score [M]
Glanular Coronal sulcus Mid or distal shaft Proximal shaft
Shaft score [S]
No chordee Mild < 30 degree chordee Moderate ( 30-60 degree) chordee Severe >60 degree chordee

Inguinal hernia, hydrocele, and cryptorchidism are the malformations most frequently linked with hypospadias. Inguinal hernia and/or hydrocele are up to 16% more prevalent [26]. Approximately 7% of individuals with hypospadias have cryptorchidism. With more proximal hypospadias, this jumps to approximately 10% [27]. Further diagnostic testing is recommended, such as an ultrasound of the urinary system and inner genital organs, to identify other nephro-urological anomalies [28]. Up to 14% of all hypospadias and up to half of the perineal hypospadias have a Müllerian remnant, resulting in catheterization difficulties, urinary blockage, or urinary tract infections (UTIs) following repair [29]. The majority of them are seen by ultrasonography. The American Urology Association cryptorchidism guideline suggests that all boys with unilateral or bilateral undescended testes and severe proximal hypospadias receive further testing to rule out a disorder of sexual differentiation (DSD), which is significantly more common in these situations.

Assessment and management

The primary objective of hypospadias treatment is to restore both aesthetic and functional normalcy. Indications for correcting hypospadias comprise spraying of urine stream, inability to pee in a standing posture, curvature causing difficulties during intercourse, reproductive concerns due to trouble sperm deposition, and decreased pleasure with genital appearance [30].

The objectives of surgical repair in males with hypospadias comprise restoration of penile curvature to guarantee long, straight arousal, the extension of the urethra to enable proper flow of urine and sperm through the glans; and the development of an aesthetically normal penis. The surgeon must evaluate the defect's possible long-term importance and have an informed debate with the boy's parents about whether surgical intervention should be undertaken. In circumstances when the penis is straight when upright and the urethral opening is sufficiently distant to permit urination while standing, a repair may be of minimal value. To guarantee a satisfactory long-term outcome, continuing into maturity, repair should be performed with the fewest possible operations. This objective is attained by preparing the patient and family for the appropriate surgery, doing an accurate anatomic evaluation, and engaging in an open dialogue regarding the functional outcome and potential consequences.

Surgical timing is crucial. The timeframe of the repair should take into account the potential unfavorable psychological consequences of surgery, the anesthetic risk to the kid, the degree of penile growth that will assist a satisfactory repair, and the age-related changes in wound healing in boys [31]. The onset of genital awareness occurs at 18 months of life and increases with age [32]. Boys who had repair sooner (typically before 12 months of age) expressed less anxiety and had better psychosexual outcomes than boys who underwent repair later [33]. Boys who get corrective surgery at a younger age may also experience fewer problems, a result that underscores the need for early intervention [33]. In comparison, adult hypospadias surgery may be associated with a greater risk of complications [34]. In 1996, based on this research, the American Academy of Pediatrics Section on Urology advised that surgical intervention for hypospadias repairs be performed between both the ages of six and 12 months, with some exceptions in our current practice [35]. Given the seriousness and the necessity for numerous treatments, some standards place the best age for hypospadias correction within six and 18 months [30]. Those who did not recollect the operation were more likely to have a better body image and be content with their overall physical appearance. These findings relate to early-life surgery to reduce psychological load.

Aesthetic hazards, age-dependent tissue diameters, and emotional repercussions of genital surgery are all factors that have an impact [28]. When considering surgery for their young boy, many parents inquire about the appropriateness of anesthesia. In the last decade, disturbing discoveries about aesthetic-induced neurotoxicity in the growing central nervous system of rats have been reported. However, scientific concerns cast doubt on the applicability of these findings to people [36]. At two years of age, neurodevelopmental impairments were not detected in children subjected to anesthesia for hernia surgery, whether it was general anesthesia or regional anesthesia [37].

Therefore, the preoperative surgical evaluation with the boy's parents must include a thorough evaluation of the advantages of surgical repair against an age-appropriate explanation of the risks of general anesthesia.

Some anatomical characteristics, such as a short glans width and a thin urethral plate, are associated with greater postoperative problems and provide technical difficulty [38,39]. However, penile size is rarely considered a consideration in determining the ideal timing for hypospadias treatment, as penile development is minimal throughout the first few years of life. Therefore, delaying surgery appears to be without benefit [28].

In hypospadias surgery, the use of preoperative androgen stimulation is contentious. Some surgeons suggest testosterone supplementation for increasing anatomical proportions. Preoperative androgen stimulation in the form of dihydrotestosterone (DHT), human chorionic gonadotropin (hCG), or testosterone can be utilized to enhance the size of the glans and penis in preadolescent males [40,41]. It is believed that increasing glans size will reduce stress on the glansplasty and improve the amount of tissue accessible for urethroplasty, hence minimizing the risk of complications. Concerns associated with androgen stimulation in these boys involve abusive tendencies and behavior, enhanced erections, skin pigmentation, and secondary masculine characteristics. All traits are temporary and dissolve spontaneously, approximately six months following the final dosage [41]. Some surgeons omit preoperative testosterone as a consequence of the perceived greater risk of bleeding and enhanced angiogenesis. Others argue that the poor healing process may be attributable to subsequent androgen administration [42].

With more than 300 restorative surgical treatments documented in the present literature, it appears that a general strategy for hypospadias surgical correction is needed [43,44]. A reoperation rate of less than 5% is considered a good indicator of success. Hypospadias complications can occur in 5-10% of patients with mild variants and 15-56% of patients with severe forms, according to most estimates over the short term [3]. Short-term outcomes may not accurately represent the experiences of males throughout their adolescence. An accurate assessment of the long-term aesthetic and functional outcomes of the repaired penis cannot be made during a 12-month follow-up following surgery because psychosexual development and pubertal physical changes have not been completed [45,46].

Using magnification, atraumatic tissue manipulation, delicate equipment, suture materials, and proper hemostasis are the most fundamental prerequisites. In most cases, the anterior and middle hypospadias is corrected in a single procedure. On the other hand, a two-step treatment is frequently required for the posterior variant [3,28].

Intraoperative Assessment

Anesthesia does not signal the end of preoperative planning. Following antiseptic preparation and intravenous antibiotic treatment, the genitalia is scrutinized to decide the surgical strategy. Except for extremely severe cases of proximal hypospadias or subsequent surgical interventions, we do not perform cystoscopies on a normal basis. The preoperative evaluation of hypospadias should continue as described. The placement of the urethral meatus, the quality of the ventral shaft tissue, and the level of penile curvature are evaluated while the kid is sleeping. Depending on the extent of penile curvature, a circumferential incision is subsequently created, and the penis is partially or entirely degloved. Care must be taken to generate a mucosal collar by rotating inner glossy preputial tissue from the dorsolateral skin to the ventrum, where it is absent. This will help with ventral shaft skin covering and produce a more aesthetically pleasing outcome [47].

Penile Curvature: Diagnosis and Treatment

Whether or not hypospadias is present, a curved penile structure (chordee) may develop. The degree of curvature is a crucial factor in deciding between a one-stage and two-stage correction. The choice to treat men's scoliosis is based on their possible functional and aesthetic difficulties as they age into adulthood. Males suffering from untreated congenital curvature or Peyronie disease have been found to experience severe morbidity at even 20-30 degrees of ventral curvature, including difficulty with intercourse and patient displeasure with the look of the penis [48]. Curvature can be caused by reduced ventral skin, a small urethra, or the inherent curvature of the erectile body. Outside of surgery, it is exceedingly difficult to determine the source of curvature. The conclusive diagnosis is made with a simulated erection in the operating theatre after the penis has been degloved. Parents should be queried whether they see a history of penile curvature during erections and may even record this in their children with photographs. Before cutting the skin, the extent of curvature must be evaluated in the operating room. Through the insertion of a catheter into the meatus, the condition of the urethra and ventral skin may be determined. To remove dysplastic dartos tissue, a circumferential incision is created and the penis is degloved beyond the penoscrotal junction. Then, a mechanical erection should be conducted, often with a tourniquet inserted at the penoscrotal junction and a sterile normal saline injection [49]. Alternately, the surgeon can squeeze the corpora at the base of the penis to mimic an erection in tiny boys without the use of injections. In addition to saline injection, prostaglandin injection can be used to generate an erection [50]. Various approaches, such as unassisted visual examination and goniometry, which works as a protractor to reliably quantify the extent of penile curvature, are used to determine the degree of penile curvature. Other technological alternatives, such as tablets and applications, are beginning to appear.

Although there is no consensus about the treatment of particular degrees of curvature, the majority of surgeons appear to think that a dorsal plication is adequate for curvatures less than 30 degrees [51]. If the curvature is greater than 30 degrees, the urethra would need to be divided. A corporal curvature higher than 30 degrees at this point necessitates a corporal lengthening surgery that involves transection of the corpus spongiosum distal to the urethra or urethra transection [52]. As these males advance through puberty and experience more considerable penile development, their curvature may increase. Therefore, it is essential to diagnose and fix curvature during the first repair [53].

Distal Hypospadias Repair

Repair of distal hypospadias is one of the most frequent surgical operations performed by pediatric urologists, and several surgical approaches have been devised to treat this condition [47]. Different procedures are used to treat this condition.

There are a variety of repair operations that may be divided into advancement, tubularization, or the use of grafting and flap surgeries. Here, we are going to discuss the most commonly used surgical techniques in treating hypospadias.

The recommended surgical procedures for hypospadias correction may vary depending on the location of the meatus. Techniques such as the tabularized incised plate (TIP) urethroplasty, the Mathieu method, the meatal advancement and glanuloplasty incorporated (MAGPI), and the glans approximation procedure (GAP) are utilized to treat distal hypospadias.

It is possible to reconstruct the urethra in a single step or two. When feasible, the majority of surgeons now choose a single-stage operation. A single-stage technique is suitable for distal, mid-shaft, and proximal hypospadias without substantial chordee. When a single operation would not be adequate to correct a severe or perineal case of hypospadias with chordee, or when performing a difficult revision hypospadias surgery, a two-stage procedure may be necessary. The preponderance of surgeons now favors tubularization of the urethral plate as a one-step procedure [51].

The most prevalent single-stage technique is a Duplay-type operation with tubularization, with or without the vertical incision in the urethral plate, as described by Snodgrass [54].

The Thiersch-Duplay (TD) Repair

The Thiersch-Duplay (TD) repair, pioneered by Thiersch and later Duplay approximately 140 years ago, employs the brilliant notion of urethral tubularization of surrounding tissues distal to the misplaced meatus [55]. They completed their repair by producing a U-shaped incision from the penile shaft using vascularized skin and extending the meatus to the coronal edge. Later, for distant hypospadias, the restoration was covered with two layers of preputial skin [56]. This procedure comprises de-epithelialization of excess preputial skin and fastening across the repair to give a blood supply replacement. The next logical step was to stretch these U-incisions into the distal glans, tabularizing the glans itself over the repair, and providing a more aesthetically pleasing meatus at the penis tip [57]. The TD method requires a glans of sufficient width to accommodate a properly sized neourethral canal, at least one water-resistant layer, and glans flaps that may approximate over the repair. Parallel incisions are made 12 Fr in diameter lateral to the glans groove; the glans wings should be fully and extensively mobilized to enable tension-free covering. Under optical magnification, a dual running subcuticular suture is used to conduct neourethral reconstruction. If the child is circumcised, a de-epithelialized pedicle flap is harvested from the preputial tissue or the more proximal axis and placed over the complete neourethral restoration [58]. If the repair is more proximal, a double dartos flap can be obtained from the dorsal prepuce, with one flap running distally and the other flap running proximally. The circumcision defect is completed by approximating the glans wings into two layers (spongiosum and then epithelium), accompanied by the mucosal collar.

The Tabularized Incised Urethroplasty (TIP)

The TIP method, a variation of the TD, is a global standard surgical treatment for hypospadias. It was originally described in 1994 by Warren Snodgrass [59]. The surgical techniques are described below. A straight 8F sound is sent into the hypospadias meatus to evaluate skin covering across the urethra. In distal hypospadias, a demarcating incision is performed 2 mm proximal to the meatus, although a U-shaped incision may be prolonged proximally to healthy skin if necessary. Degloving the penis to the penoscrotal union. In every situation, an artificial erection is performed, as even coronal hypospadias is occasionally coupled with penile bending. If a minor chordee remains following skin release, dorsal plication is performed to rectify the corpora cavernosa's asymmetry. The tunica albuginea is incised longitudinally on either end just lateral to the neurovascular bundle opposing the point of curvature, followed by the placement of 6-0 Prolene sutures with the knots concealed. There is no need for substantial mobilization of the neurovascular bundle while performing dorsal plication. Next, 1:100,000 epinephrine is injected into the ventral glans at the visible intersection of the glans wings and urethral plate. Then, parallel incisions are made to detach the plate from the glans, and the glans wings are deployed laterally. Depending on its native groove, the plate is just 4 to 8 mm broad at this point. A linear relaxing incision is created from the inside of the meatus to the distal edge of the plate. This incision penetrates the epithelial surface of the plate and spreads deeper into the connective tissues underneath, reaching the corpus cavernosum. With the surgeon and helper maintaining counter-traction with tiny forceps, the plate is observed to be considerably widened upon division until further incisions offer no more mobility. Rather than a knife, tenotomy shears are indicated for this procedure so that an appropriate depth may be achieved without harming the corpus cavernosum. When the urethral plate is naturally grooved, the incision will be shallower than when the plate is naturally flat. Some surgeons perform the relaxing incision first, followed by parallel incisions to establish the plate's breadth. Despite this, this procedure regularly expands the plate to 13 to 16 mm, independent of its arrangement, assuring that the neourethra will be larger than 12F. If bleeding develops, epinephrine diluted 1:1000 is poured over the incision, and pressure is maintained for many minutes. If a tourniquet is required, it might be placed near the base of the penis. Electrocautery shouldn't be used to make holes in the plate or stop bleeding so that the plate's tissues and the corpora cavernosa underneath don't get hurt.

Next, a 6F stent is inserted into the bladder for urine diversion following surgery. The urethral plate is subsequently tabularized. To guarantee that the neo-meatus has a wide oval aperture, the initial stitch is always put at the level of the mid-glans, and no more than one or two stitches are removed distally. In this procedure, a single layer of 7-0 chromic catgut suture of full thickness is used. Those who prefer suture materials with a slower absorption rate might try subcuticular closures.

A thin dartos pedicle derived from the dorsal prepuce and shaft skin covers the whole neourethra. Glansplasty is then performed, commencing at the cornea and extending distally for a total of three stitches. Even though tiny sutures at the four and eight o'clock locations may evert the meatus somewhat for cosmetic purposes, securing the neourethra to the glans is not essential. The mucosal collar is approached in the midline, and the skin of the shaft is remodeled to resemble the median raphe. Subcuticular sutures are employed to avoid the suture tracts previously observed when 6-0 chromic catgut was put through the skin. After applying a dressing, the child is sent home [54].

Flap Methods

The Mathiew procedure is based on a meatal flap. This operation was documented for the first time in 1932, but it appears to have been performed earlier. The Mathieu method does not begin with penis degloving; rather, a penile shaft tissue flap is used to generate the neo-urethra. The Mathieu technique begins by determining the extent of the urethral gap from the meatus to the tip of the glans. Along the urethral plate, an equivalent distance is traced on the proximal penile shaft skin. An incision is created along these lines. For the proximal flap, an acceptable width of 7 to 8 mm is measured, with this width tapering to 5 to 6 mm towards the distal limit of the glans. After skin and glanular incisions, the shaft skin is degloved. The underlying tissue of the flap is dissected with care, enabling the flap to be advanced to the top of the glans. The flap is rolled over at the meatus and approximated to the lateral borders of the urethral plate with a running suture. Meatus has reached full maturity. The sutures are covered with a dartos flap of tissue, the glans wings are approached, and then a typical circumferential closure is done [60]. Concerns arise surrounding the vasculature of the utilized flap; if the flap's base is not adequately wide, the blood supply may be disrupted, hence increasing the prospect of fistula and stenosis. Others have expressed alarm at the fish-mouth look of the meatus. This method has been upgraded to the slit-like adjusted Mathieu (SLAM) process, which has shown favorable results, including an enhanced look of the meatus [61].

Advancement Techniques

Advancement methods do not necessitate tubularization of the urethral plate and are usually reserved for the most distal glanular meatus with minor penile curvature. Urethromeatoplasty employs the Heineke-Mikulicz concept, in which a longitudinal, vertical incision is made in the ectopic meatus and, subsequently, its margins are closed horizontally. This provides a cosmetically normal meatus and straightens the posterior urethral plate. This approach is especially beneficial in the presence of a stenotic, distal meatus with an accompanying blind-ending pit in the middle of a closed glans. The meatal advancement glanuloplasty would become one of the most often performed procedures to treat glanular hypospadias (MAGPI). The primary purpose of this operation is to distally advance the meatus without technically tabularizing the urethra [62]. The frequency of problems reported following the MAGPI technique complications occurs up to 10% [63]. Meatal stenosis and meatal regression are the most commonly encountered issues, while other uncommon complications consist of urethro-cutaneous fistulas and chordee.

The Glans Approximation Procedure (GAP)

The glans approximation method is a surgical approach developed for individuals with proximal glanular/coronal hypospadias who have a broad, steep glanular groove and a non-compliant or fish-mouth meatus, which is frequently found in the mega-meatus intact prepuce type [64].

Proximal Hypospadias Repair

The treatment of severe hypospadias has proven contentious. This disagreement persists as to the optimal treatment for proximal hypospadias. Numerous hypospadias correction procedures have been published, reflecting the difficulties of achieving optimal surgical outcomes for this illness [65]. Even though one-stage surgery has been shown to work for some types of proximal hypospadias, many people still prefer the more traditional two-stage method when moderate to severe chordee is present so that the length of the penis can be straightened during the first-stage repair.

One-stage proximal hypospadias correction often entails dorsal plication to restore ventral penile curvature and is one of many urethroplasty procedures. These can be differentiated according to the tissue employed in the repair, namely preputial skin, local skin, and buccal transplant. The preputial island flap is widely recognized as an innovation that Duckett contributed to [66]. In this procedure, the inner prepuce is elevated as a pedicle flap, translated ventrally, and used as an Onlay graft to cover the urethral plate following degloving the penis and straightening the chordee. Neo-urethras have a roof made up of the urethral plate. To prevent stricture development, the onlay excludes circular anastomosis. The inner prepuce is similarly employed as a pedicle flap in the Asopa variant of the technique, but the neo-urethra is left connected to the underside of the foreskin. Consequently, the skin and neo-urethra share a blood supply [67]. Higher complication rates were observed in the Duckett technique, and those included poor aesthetic results marked by excessive ventral bulkiness, penile torsion, and meatal anomalies; fistulas, strictures, total breakdown, and anterior urethral diverticuli formation [68].

The two-stage repair has been the preferred method of most surgeons for treating proximal hypospadias since the treatment of severe ventral penile curvature has shifted toward corporal lengthening techniques. Modern two-stage methods may be broadly classified, despite their many technical variants, into repair with free graft or repair with pedicle flap.

The Bracka two-stage repair is a urethroplasty technique that employs a free graft taken from the inner preputial skin or buccal mucosa [69]. STAG is an adaptation of Bracka's initial explanation [70]. In the first step, the penile curvature and urethral plate are rectified. A graft receiving bed is created by extending a midline incision into the glans. On the ventral penile shaft, compressive packing and patterning of the graft can reduce hematoma development and enhance graft uptake. Six months later, a U-shaped incision identical to the Thiersch-Duplay method is created, the urethra is tabularized, and glansplasty is carried out. Layered closure is performed to preserve vascular flow to promote healing [69]. The Byars flap treatment employs extra dorsal preputial skin, which is transferred ventrally with its vascular pedicle during the first surgery, as the urethral scaffold [71]. In the ventral part of the penis, the skin can be connected in the midline or positioned as a single unit, as in the STAG repair. In the second step, the neourethra is sealed by making a large U-shaped incision with a typical Thiersch-Duplay glansplasty. The development of a waterproof, two-layer closure and the establishment of a lumen of uniform diameter along the course of the urethroplasty are important technical elements. To guarantee that the neourethra retains a sufficient blood supply, several phases of closure are necessary. In particular, making a soft dartos bed above the clitoroplasty in the first step will ensure enough blood flow for the urethroplasty in the second step.

Regardless of the methodology, it is essential to evaluate the quality of the graft or flap during the second phase of the surgery. As an interim step, if skin deficit or tethering prevents safe closure, a dorsal inlay buccal mucosal transplant may be employed as an interim measure [72]. After graft harvesting, the urethra is rebuilt when all of the tissues are pliable. Alternately, the second step of repair can be performed simultaneously with a dorsal buccal graft inlay and a urethroplasty. It is essential to check that the penile curvature is rectified with a subsequent synthetic erection before urethroplasty. If needed, a dorsal plication or repeat corporal lengthening can be done to fix a slight curvature that keeps coming back.

Postoperative complication

The majority of early postoperative problems are caused by incorrect surgical techniques and may be readily avoided via improved procedure planning and tissue management. These problems include edema, hematoma development, wound dehiscence, flap decay, and fistula formation [73]. To prevent hematoma development, optimal hemostasis must be achieved. As previously stated, adequate tissue manipulation is required to prevent postoperative edema. A compression circumferential covering can also reduce postoperative edema.

Long-term outcomes

There is a dearth of consistency in the literature when it comes to hypospadias correction procedures, as well as standardized definitions of problems and methods for evaluating outcomes [74]. Many questionnaires have been devised to evaluate the results of hypospadias treatment. Each questionnaire has its pros and limitations. These include the (Pediatric) Penile Perception Score (PPPS), the (Hypoplasia) Objective Scoring System, the (PedsQl), and the Hypoplasia Objective Penile Evaluation Score (HOPE) [75,76].

More than 70% of all patients who have hypospadias treatment are deemed cosmetically pleasing. More than 80% of males with repaired hypospadias had good sexual function [77]. However, these individuals are frequently prevented from initiating sexual interaction and frequently fear mockery due to the look of their genitals [77,78]. Symptoms of the lower urinary tract were twice as prevalent in individuals who had had hypospadias correction compared to controls [77]. After tabularized incised plate (TIP) urethroplasty, an obstructive urine flow pattern is usually observed, which may be due to aberrant elastic properties of the produced tube [79]. Almost 39% of patients who underwent proximal hypospadias surgery showed voiding problems, including hesitation and spraying [77]. Urinary problems (e.g., meatal stenosis, fistula, or urethral stenosis) may emerge years after the initial surgery; consequently, long-term follow-up is required [80].

## Conclusions

Hypospadias is a frequent disorder with an unknown cause and a wide range of manifestations and degrees of severity. The objective of hypospadias restoration is to restore normal function and appearance. The hypospadias is often repaired between six and 18 months of age. The optimal age for surgical intervention is still a matter of controversy and is impacted by anesthetic risks, tissue size at various ages, postoperative problems, and psychosocial effects. Long-term results for both function and appearance are typically satisfactory, although still inferior to those of males without hypospadias. Various procedures for its surgical intervention have been documented. Functional results are enhanced by early rebuilding. It has been shown that around 25% of people with hypospadias require a second procedure. This disorder is most effectively treated by a multidisciplinary team comprising of a urologist, neonatologist, pediatric surgeon, reconstructive surgeon, endocrinologist, geneticist, nurse, and mental health counselor. A thorough inspection of the genitalia should be performed at birth and, of course, before circumcision is planned.
